# The role of ARL4C in predicting prognosis and immunotherapy drug susceptibility in pan-cancer analysis

**DOI:** 10.3389/fphar.2023.1288492

**Published:** 2023-12-20

**Authors:** Hanshu Zhao, Kaiqi Yang, Ziqi Yue, Ziyin Chen, Zhe Cheng, Hongcheng Sun, Changze Song

**Affiliations:** ^1^ Scientific Research Center, The Seventh Affiliated Hospital, Sun Yat-sen University, Shenzhen, China; ^2^ Department of Neurology, The First Affiliated Hospital of Harbin Medical University, Harbin, China; ^3^ Clinical Medicine, Harbin Medical University, Harbin, China; ^4^ Department of Forensic Medicine, Harbin Medical University, Harbin, China; ^5^ Department of Urology, China-Japan Friendship Hospital, Beijing, China; ^6^ Department of Gastroenterology, The First Affiliated Hospital of Harbin Medical University, Harbin, China; ^7^ Department of Urology, The Fourth Hospital of Harbin Medical University, Harbin, China

**Keywords:** ARL4C, pan-cancer, tumor microenvironment, prognosis, immunotherapy drug susceptibility

## Abstract

**Background:** ARLs, which are a class of small GTP-binding proteins, play a crucial role in facilitating tumor tumorigenesis and development. ARL4C, a vital member of the ARLs family, has been implicated in the progression of tumors, metastatic dissemination, and development of resistance to therapeutic drugs. Nevertheless, the precise functional mechanisms of ARL4C concerning tumor prognosis and immunotherapy drug susceptibility remain elusive.

**Methods:** By combining the GTEx and TCGA databases, the presence of ARL4C was examined in 33 various types of cancer. Immunohistochemistry and immunofluorescence staining techniques were utilized to confirm the expression of ARL4C in particular tumor tissues. Furthermore, the ESTIMATE algorithm and TIMER2.0 database were utilized to analyze the tumor microenvironment and immune infiltration associated with ARL4C. The TISCH platform facilitated the utilization of single-cell RNA-seq datasets for further analysis. ARL4C-related immune escape was investigated using the TISMO tool. Lastly, drug sensitivity analysis was conducted to assess the sensitivity of different types of tumors to compounds based on the varying levels of ARL4C expression.

**Results:** The study found that ARL4C was highly expressed in 23 different types of cancer. Moreover, the presence of high ARL4C expression was found to be associated with a poor prognosis in BLCA, COAD, KIRP, LGG, and UCEC. Notably, ARL4C was also expressed in immune cells, and its high expression was found to be correlated with cancer immune activation. Most importantly, the drug sensitivity analysis revealed a positive correlation between ARL4C expression and the heightened sensitivity of tumors to Staurosporine, Midostaurin, and Nelarabine.

**Conclusion:** The findings from our study indicate that the expression level of ARL4C may exert an influence on cancer development, prognosis, and susceptibility to immunotherapy drugs. In addition, the involvement of ARL4C in the tumor immune microenvironment has expanded the concept of ARL4C-targeted immunotherapy.

## 1 Introduction

Cancers, being malignant diseases with significant implications for human health and quality of life (Torre et al., 2015; Collaborators, 2022; Kong et al., 2022), have prompted the exploration of diverse treatment approaches beyond conventional options like surgery, radiotherapy, and chemotherapy (Nia et al., 2020; Sancar and Van Gelder, 2021). In contemporary times, immunotherapy has emerged as a new and innovative alternative to anti-tumor therapy (Yin et al., 2023). However, the effectiveness of the majority of FDA-approved treatments, such as anti-PD-1/PD-L1 immunotherapy, currently stands at a mere 10%–25% (Zhang et al., 2021). Consequently, researchers are actively exploring novel immunotherapeutic targets to enhance the prognosis of individuals afflicted with malignancies.

ADP-ribosylation factor-like protein 4C (ARL4C), as ARL7 of the ARF/ARL (ADP-ribosylation factor/ARF-like protein) family, is a 192-amino-acid GTP enzyme on the cell membrane (Liao et al., 2020). ARL4C plays a significant role in various physiological and pathological processes, including cellular morphological alterations, microtubule dynamics, cholesterol secretion, vesicle transport, and signal transduction including RAS/MAPK and Wnt/β-catenin signaling pathways (Fujii et al., 2022). ARL4C plays an important role in tumorigenesis and also serves as a prognostic biomarker in glioblastoma, renal cell carcinoma, gastric, ovarian, lung and colorectal cancers (Fujii et al., 2015; Chen et al., 2016; Hu et al., 2018; Chen et al., 2019; Isono et al., 2019; Wakinoue et al., 2019). Additionally, Liao et al. indicate that ARL4C plays an important role in the resistance of non-small cell lung cancer (NSCLC) cells to Erlotinib, and the regulation of the drug resistance of lung cancer cells by ARL4C was through activating the β*-*catenin/JAK2/STAT5A signaling pathway (Liao et al., 2020). Therefore, ARL4C may serve as a potential therapeutic target for overcoming resistance to other types of anti-tumor drugs and needs to be further explored.

Tumorigenesis is not only determined by the intrinsic properties of cancer cells but also by their interactions with components of the tumor microenvironment (TME). TME is a complex cellular ecosystem, jointly formed by leukocytes, fibroblasts, and vascular endothelial cells, playing a crucial role in tumor progression (Yang et al., 2020). It has a critical role in determining tumor progression, antitumor immunity, and the response to immunotherapy (Nel et al., 2022). Currently, cancer immunotherapy has made significant strides in overcoming numerous challenges in cancer treatment. Nonetheless, it is crucial to acknowledge that immunotherapy is not without its limitations, and the tumor microenvironment (TME) poses a substantial barrier to the efficacy of cancer treatment and the clinical success of immunotherapy (Devalaraja et al., 2020). The research has identified that ARL4C is expressed in macrophages and is a direct transcriptional target of LXR in macrophages. Tumor-associated macrophages (TAMs) are a major component of the TME (Lopez-Yrigoyen et al., 2021). ARL4C has been shown to be highly expressed in various tumor cells. However, whether ARL4C is associated with the immune system and tumor immune microenvironment has not been studied.

Consequently, we conducted a bioinformatics analysis to investigate the involvement of ARL4C in cancer development, examining its expression patterns, prognostic implications, and association with the immune microenvironment across various cancer types. Additionally, we explored the relationship between ARL4C and drug susceptibility, aiming to uncover novel approaches for ARL4C-targeted immunotherapy.

## 2 Materials and methods

### 2.1 Source and processing of the expression data of ARL4C

The expression data (log2 TPM+1) of the ARL4C gene in the TCGA (The Cancer Genome Atlas) tumor database was analyzed by UCSC Xena, along with the corresponding clinical information (Han et al., 2022). Information regarding ARL4C expression in normal tissues was obtained from GTEx (TPM). Furthermore, a comprehensive analysis of ARL4C expression in cancer cell lines was conducted using the CCLE database (Yang et al., 2022). The data analysis was carried out utilizing R software (Version 4.2.0), and the “ggpubr” R package was employed to generate radar or box plots.

### 2.2 Immunohistochemical and immunofluorescence staining

The Department of Pathology at The Fourth Hospital of Harbin Medical University provided paraffin sections of tissue specimens of specific tumors and corresponding para-cancer specimens for immunohistochemistry (IHC) and immunofluorescence (IF) analysis. The ARL4C antibody (10202-1-AP) was obtained from Proteintech, the CD206 antibody (Ab64693) was acquired from Abcam, and the α-SMA antibody (GB111364) was purchased from Servicebio. The calculation of IHC density involved determining the ratio between the intensity of BCA and the size of the area, whereas the IF density was determined by the ratio between the fluorescence intensity of positive cells and that of DAPI. This study received approval from the Institutional Research Ethics Committee of the Fourth Affiliated Hospital of Harbin Medical University.

### 2.3 Genetic variation analysis of ARL4C genome

The cbioportal database (www.cbioportal.org) was utilized to conduct a comprehensive analysis of genomic alterations in specific genes. The visualization of genomic alterations in ARL4C across 32 TCGA cancer types was achieved through the utilization of “Cancer Types Summary” and “Cancer Type” tools. The frequency of ARL4C copy number alterations and mutations in TCGA tumors was observed. Additionally, HM450 (HumanMethylation450) methylation data for each tumor were acquired from the GSCA (Generalized Structured Component Analysis, http://bioinfo.life.hust.edu.cn/GSCA/#/) database and UCLCAN (http://ualcan.path.uab.edu/index.html) database. The relationship between ARL4C expression levels and its promoter methylation levels was examined and graphically represented using the R package “ggpubr”.

### 2.4 Prognostic characterization of ARL4C

Based on overall survival (OS) data obtained from TCGA, an investigation was conducted to determine the prognostic characteristics of ARL4C. The analysis involved examining the relationship between ARL4C expression and patient outcomes, with patients being classified into high and low-expressing ARL4C subgroups based on their median expression levels. The survival data for each type of cancer was subjected to Kaplan-Meier survival and logrank tests, and the resulting survival curves were generated using the R packages “survminer” and “survival.” Furthermore, univariate Cox models were utilized to evaluate the association between ARL4C expression and patient outcomes. The single gene logistic regression analysis was conducted using the “stats (4.2.1)” R package, utilizing data from the TCGA database. The PrognoScan database (http://www.abren.net/PrognoScan/) analyzed the correlation of ARL4C expression and survival including OS and disease-free survival (DFS). Statistical significance was determined by a *p*-value of less than 0.05.

### 2.5 Analysis of the TME and immune cell infiltration in relation to ARL4C

The ESTIMATE algorithm was employed to calculate stromal or immune cell scores, indicating the abundance of stromal and immune cells (Pender et al., 2021). The significance of the corresponding component in the TME increases as the score rises. ARL4C expression, ImmuneScore, and StromalScore were obtained for the tumor using the “ESTIMATE” R package and Spearman correlation analysis. The TIMER2.0 database (http://timer/cistrome.org) and the cibersort algorithm were utilized for immune cell infiltration correlation analysis (Meng et al., 2022). Furthermore, the classification of ARL4C high and low expression groups and the assessment of immune cell infiltration were conducted using ssGSEA (single sample Gene Set Enrichment Analysis).

The immune filtration analysis and correlation analysis were conducted using the TCGA database to examine the relationship between ARL4C expression in liver hepatocellular carcinoma (LIHC) and the biomarkers CD206 (indicative of M2 macrophages) and α-SMA (indicative of cancer-associated fibroblasts). The statistical data were obtained through the utilization of the “ggplot2 (3.3.6)” R package and Spearman correlation analysis.

### 2.6 ARL4C-associated single-cell analysis

Single-cell analysis was performed using TISCH (Tumor Immunization Single Cell Hub), which was visually represented through a heat map, scatter plot, and violin plot (Sun et al., 2021). The data collection, processing, and cell annotation procedures are detailed in the documentation section of the TISCH website (http://tisch.comp-genomics.org/documentation/). The basal cancer immunotherapy dataset is denoted as GSE123813 (droplet-based 5′-scRNA- and TCR-seq libraries from 11 patients with advanced BCC before and after anti-PD-1 treatment in site-matched primary tumors), the kidney cancer immunotherapy dataset as GSE145281 (Single-cell RNA-seq of baseline pretreatment PBMC samples from 10 cancer patients with 5 responders and 5 nonresponders), and the immunotherapy dataset for cutaneous Merkel cell carcinoma as GSE117988 (two patients with metastatic Merkel cell carcinoma with autologous Merkel cell polyomavirus specific CD8^+^ T cells and immune-checkpoint inhibitors).

Following this initial classification, the expression levels of the ARL4C gene were ascertained for each cell. For each distinct cell type, a Mann-Whitney U test was independently applied to contrast the distribution of ARL4C gene expression across groups of cells pre- and post-treatment, in order to evaluate the presence of statistically significant disparities. The wilcox.test function within the R programming environment facilitated the execution of these individual Mann-Whitney U analyses.

### 2.7 Enrichment analysis of ARL4C-Related gene sets

The “gmt” file (h.all.v7.4.symbols.gmt) of the marker gene sets were obtained from MSigDB (Molecular Signature Database) (Zhang et al., 2021). A total of 50 Hallmark gene sets from this file were utilized to compute normalized enrichment scores (NES) and false discovery rates (FDR) for differentially expressed genes (DEGs). The GSEA enrichment analysis was conducted using the R package “clusterProfiler”.

### 2.8 Predictive analysis of ARL4C-targeted immunotherapy

Predictive analysis of ARL4C-related immunotherapy in the mouse samples was performed using the TISMO database (Zeng et al., 2022). Additionally, the TIDE (Tumor Immune Dysfunction and Exclusion) database was employed to analyze ARL4C-associated immune escape in tumors (Ding et al., 2022). Somatic mutation data for all patients in TCGA were obtained from the UCSC Xena database, and subsequently, tumor mutation burden (TMB) scores were calculated. Spearman’s correlation coefficient was employed to examine the association between ARL4C expression and either TMB or microsatellite instability (MSI).

### 2.9 Drug sensitivity analysis

The investigation of the relationship between ARL4C expression and drug response was conducted using the CellMiner drug data (Ding et al., 2022). To predict sensitive compounds based on ARL4C expression, the GSCA database, which encompasses GDSC (Genomics of Drug Sensitivity in Cancer) and CTRP (Cancer Therapeutics Response Portal), was utilized (Liu et al., 2022).

### 2.10 Statistical analysis

The samples were divided into high and low groups based on the Median. Survival analysis was conducted using the Kaplan-Meier method, and the results were compared using a log-rank test, which provided the hazard ratio, 95% confidence interval, log-rank test, and *p*-value. The correlation between two variables was assessed using either the Spearman or Pearson test. The PrognoScan databases produced HR and *p*-values or Cox *p*-values according to a log rank test. All statistical analyses were performed using R software (Version 4.2.0). Statistical significance was determined by a *p*-value less than 0.05.

## 3 Results

### 3.1 ARL4C exhibits widespread expression in pan-cancer

**FIGURE 1:**
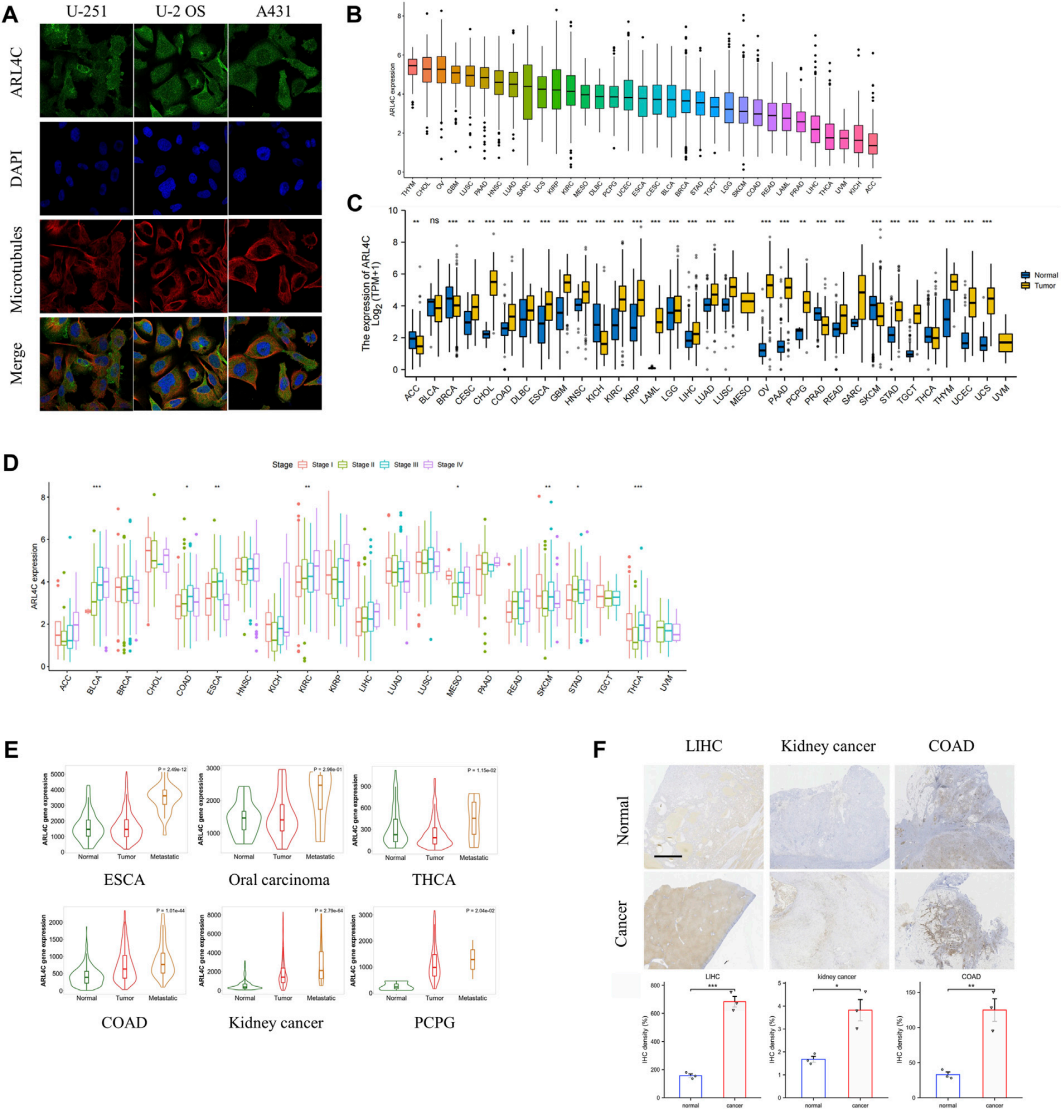
Expression of ARL4C in pan-cancer. **(A)** The distribution of ARL4C in tumor cells was examined using datasets from the Human Protein Atlas (HPA). **(B)** The expression of ARL4C was analyzed in various types of cancer using data from The Cancer Genome Atlas (TCGA). **(C)** The expression of ARL4C was analyzed between tumor and normal tissues, as determined by the Wilcoxon rank-sum test using data from the Genotype-Tissue Expression (GTEx) and TCGA databases (**p < 0.01, ***p < 0.001). **(D)** ARL4C expression was analyzed across different pathological stages, as determined by a one-way analysis of variance (ANOVA) test (*p < 0.05). **(E)** The expression of ARL4C was compared among normal tissues, primary tumors, and metastatic tumors using a one-way ANOVA test (*p < 0.05). **(F)** Representative immunohistochemistry (IHC) images of postoperative samples from patients with liver hepatocellular carcinoma (LIHC), kidney cancer, and colon adenocarcinoma (COAD) are shown (scale bar = 2.5 mm, *n* = 3).

ARL4C assumes crucial functions in the realm of tumor cell biology, encompassing stem cell-like attributes, proliferation, and resistance to pharmaceutical agents. Our investigation revealed that ARL4C is ubiquitously present in the plasma membrane and cytosol of U-251 (human glioblastoma cell lines), U-2 OS (human osteosarcoma cell line), and A431 (human skin cancer cell line), as evidenced by the HPA (Human Protein Atlas) datasets (Figure 1A). In normal tissues, the spleen, cerebellar hemisphere, and sun-exposed lower leg skin exhibited the highest expression of ARL4C, while the skeletal muscle, left ventricle, and testis displayed the lowest expression (Supplementary Figure S1). The extensive distribution of ARL4C implies its involvement in diverse biological processes. Based on the analysis of TCGA data, it was observed that ARL4C exhibited the highest expression levels in thymoma (THYM), cholangiocarcinoma (CHOL), and ovarian cancer (OV). Conversely, ARL4C expression was found to be lowest in adrenocortical carcinoma (ACC), kidney chromophobe (KICH), and uveal melanoma (UVM) (Figure 1B). Furthermore, significant differences in ARL4C expression levels between tumor and normal tissues were observed in 29 out of 33 types of cancers (*p* < 0.05, log2FC > 1) (Figure 1C). Specifically, ARL4C expression was lower in ACC, breast cancer (BRCA), KICH, prostate adenocarcinoma (PRAD), skin cutaneous melanoma (SKCM), and thyroid carcinoma (THCA) compared to normal tissues. Conversely, ARL4C expression was higher in all other 23 types of cancers compared to normal tissues. The diverse level of ARL4C expression suggests a multifaceted role for ARL4C, necessitating further investigation.

To elucidate the association between ARL4C and the progression of various cancers, we assessed the mRNA levels of ARL4C across different pathological stages in 21 different cancer types. Our findings, as depicted in Figure 1D, demonstrate significant variations in ARL4C expression among different pathological stages in 8 tumor types (*p* < 0.05, log2FC > 1). Notably, ARL4C expression exhibited an upward trend in bladder urothelial carcinoma (BLCA) and KICH with the advancement of pathological stages. In conjunction with RNA-seq and microarray data, ARL4C exhibited significantly elevated expression levels in the metastasis of esophageal carcinoma (ESCA), oral carcinoma, THCA, colon adenocarcinoma (COAD), kidney cancer, and pheochromocytoma and paraganglioma (PCPG) compared to primary tumors (Figure 1E). In order to further validate the findings, we obtained postoperative samples from liver hepatocellular carcinoma (LIHC), kidney cancer, and COAD patients for immunohistochemical analysis. Consistent with the bioinformatical analysis, the expression level of ARL4C was found to be higher in various types of cancer tissues (LIHC, kidney cancer, and COAD) compared to healthy tissues (Figure 1F). This finding aligns with previous studies that have implicated ARL4C in the malignant progression of tumors.

### 3.2 The genetic alterations and DNA methylation profile of ARL4C

**FIGURE 2:**
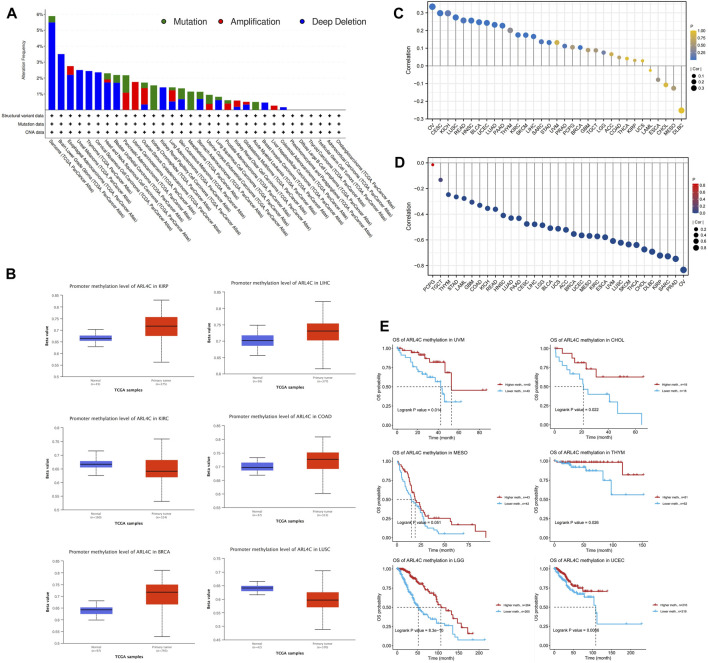
DNA methylation, genetic alterations, and associated prognosis. **(A)** Analysis of the mutations in ARL4C among cancer patients by utilizing the cBioPortal database. **(B)** Analysis of the correlation between ARL4C expression and levels of promoter methylation. **(C)** Association between ARL4C and copy number variations (CNV). **(D)** Correlation between ARL4C and methylation. **(E)** Assessment of the impact of ARL4C methylation on prognosis using the Kaplan-Meier approach and log-rank test for survival analyses.

Genomic variation plays a crucial role in the processes of tumorigenesis and evolution. Consequently, we conducted an assessment of the mutation rate of ARL4C in cancer patients using the cBioPortal database (Figure 2A). Among the various cancer types examined, sarcoma, brain lower grade glioma, and esophageal adenocarcinoma exhibited the highest frequencies of alterations, surpassing 2%, with a predominant occurrence of deep deletions. Conversely, amplification was predominantly observed in OV, uterine carcinosarcoma (UCS), and pancreatic adenocarcinoma (PAAD). The mutation counts for all cancer types are presented in Supplementary Figure S2. Given that DNA methylation typically promotes tumor initiation and proliferation, we conducted an investigation into this epigenetic modification in 33 types of cancers (Figure 2B). The results indicated that there were increased levels of methylation in the promoter region of ARL4C in BRCA, COAD, kidney renal papillary cell carcinoma (KIRP), and LIHC when compared to normal tissues. Conversely, a decrease in the methylation levels of its promoter was observed in lung squamous cell carcinoma (LUSC) and kidney renal clear cell carcinoma (KIRC).

Recognizing the significance of copy number variation (CNV) in tumor development, we further examined the relationship between ARL4C and CNV in the 33 tumors (Figure 2C). Notably, ARL4C exhibited a positive correlation with CNV in several tumors, including OV, cervical squamous cell carcinoma and endocervical adenocarcinoma (CESC), and KICH. Meanwhile, we conducted an investigation into the relationship between ARL4C and methylation, as depicted in Figure 2D. This analysis revealed a strong association between ARL4C mRNA expression and methylation in various cancer types, with the exception of PCPG. Subsequently, we examined the prognostic value of ARL4C methylation in TCGA, as illustrated in Figure 2E. Notably, high levels of ARL4C methylation were significantly correlated with improved overall survival in patients with THYM, UCEC, UVM, CHOL, and brain lower-grade glioma (LGG), indicating that ARL4C methylation may serve as a protective factor in these individuals.

### 3.3 ARL4C is identified as a prognostic factor in various cancer types, influencing tumor progression

**FIGURE 3:**
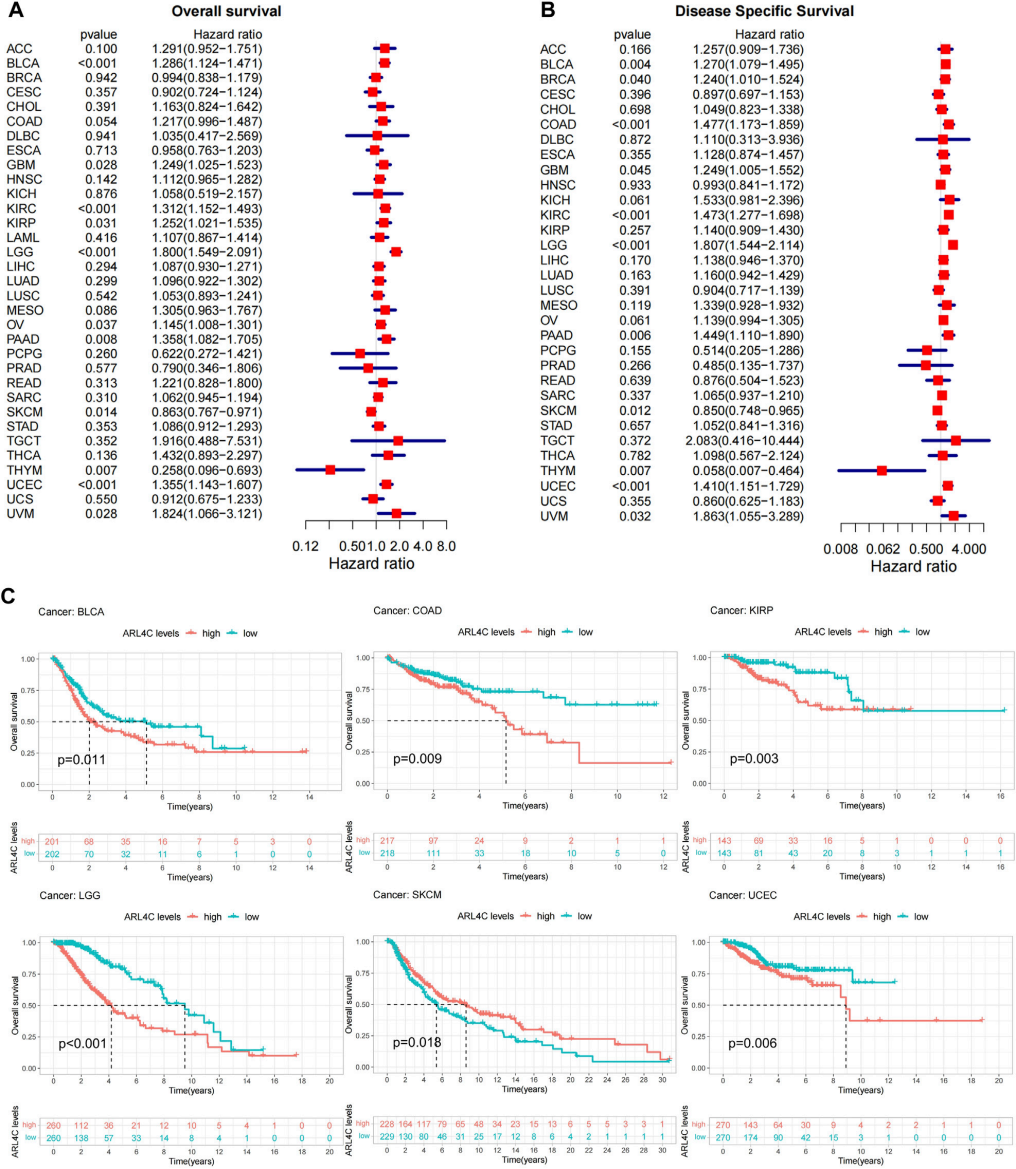
Prognostic analysis of ARL4C. **(A)** Overall survival (OS) analysis of ARL4C across 33 different cancer types **(B)** DSS analysis of ARL4C across 33 different cancer types. **(C)** ARL4C-related Kaplan-Meier curves were utilized to analyze survival outcomes using the Kaplan-Meier approach and log-rank test.

Previous studies found that high expression of ARL4C was found in low-grade glioblastoma (GBM) and was correlated with poorer progression-free survival (PFS) and overall survival (OS) in patients (Liu et al., 2022). Therefore, univariate Cox regression analysis was conducted to examine the relationship between ARL4C expression levels and OS in various cancer types. Figure 3A illustrates that ARL4C was significantly associated with hazard ratios in 11 cancer types. Among these, ARL4C was identified as a risk factor in 9 cancers (BLCA, GBM, KIRC, KIRP, LGG, OV, PAAD, UCEC, UVM), while acting as a protective factor in SKCM and THYM. Concurrently, a DSS analysis was conducted to eliminate the influence of non-cancer fatalities on OS. The results of the DSS analysis exhibited a significant level of concurrence with the OS analysis, as depicted in Figure 3B. In order to gain further insight into the impact of ARL4C on patient prognosis, a Kaplan-Meier curve analysis was performed (Figure 3C). This analysis revealed that heightened expression of ARL4C was correlated with a poorer prognosis in BLCA, COAD, KIRP, LGG, and UCEC, while conversely associated with a better prognosis in SKCM.

Furthermore, a single gene logistics regression analysis was conducted to investigate the role of ARL4C as a prognostic factor. The findings were consistent with the Kaplan-Meier curve analysis, indicating that ARL4C impacted the TNM stages in BLCA, COAD, and KIRP, as well as the clinical stage in UCEC and LGG. However, no significant association was observed in SKCM (Supplementary Table S1). Finally, we used the PrognoScan database to validate the survival outcomes. The correlations between ARL4C expression and clinical outcomes from 12 cancer types in 124 databases are displayed in Supplementary Table S2. These results suggest that ARL4C may serve as a prognostic factor in many tumor types by influencing tumor stages.

### 3.4 ARL4C is involved in immune evasion pathways in multiple cancers

**FIGURE 4:**
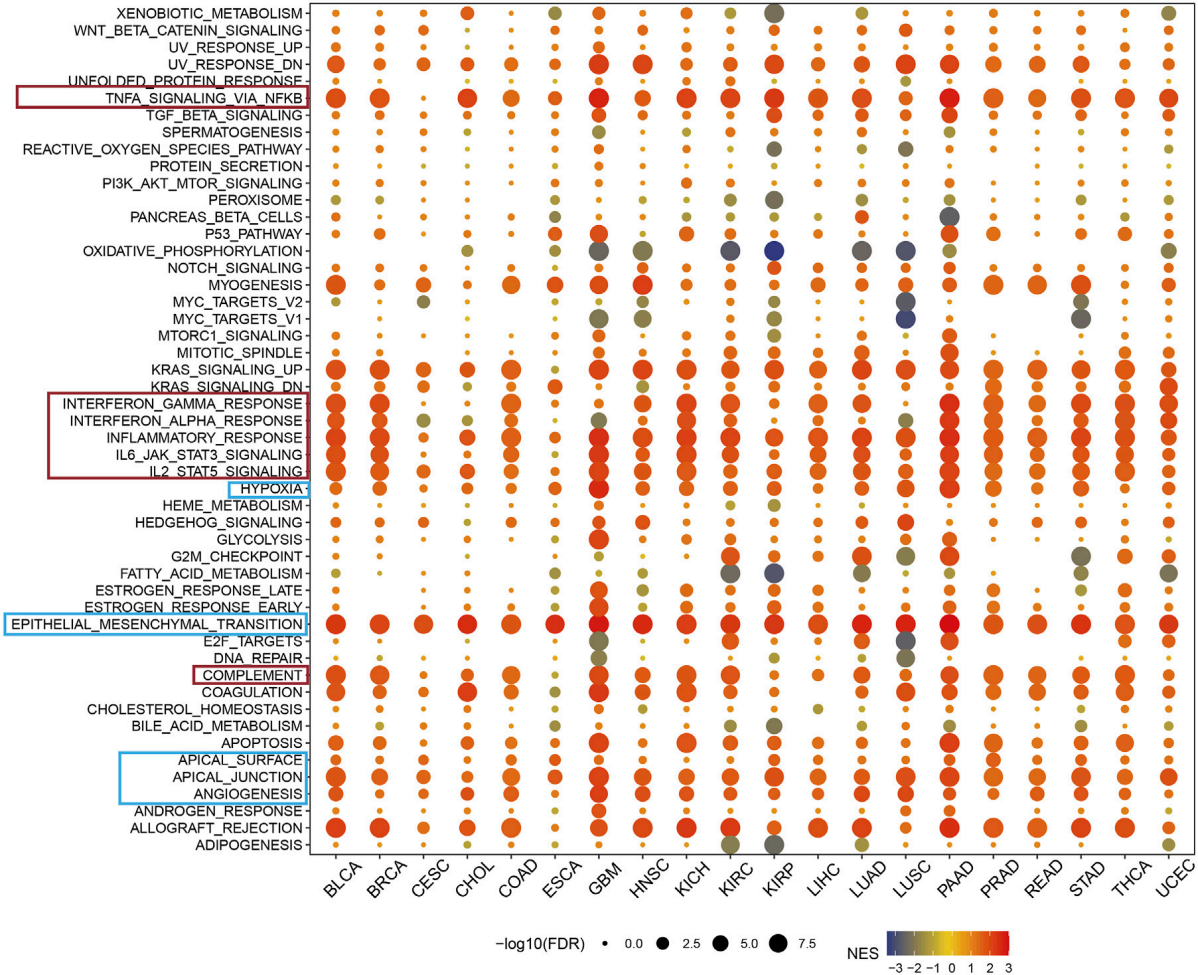
GSEA of ARL4C in pan-cancer. The red modules exhibited a strong correlation between ARL4C expression and immune signaling pathway, whereas the blue modules were associated with cell adhesion and remote metastasis pathways.

In a pan-cancer context, differentially expressed genes (DEGs) between low and high ARL4C-expressing subgroups were subjected to Gene Set Enrichment Analysis (GSEA) to identify cancer features associated with ARL4C, as illustrated in Figure 4. The expression of ARL4C exhibited a significant and positive correlation with immune-related pathways such as TNF-α/NF-κB, INF-γ, INF-α, IL-6/JAK/STAT3, and hypoxia signaling pathways, thereby establishing a connection between ARL4C and tumor immunity. Notably, the high ARL4C group demonstrated significant enrichment of epithelial-mesenchymal transition (EMT) in most tumors.

Previous research has established a notable correlation between the oxidative metabolism of tumors and the effectiveness of PD-1 immunotherapy. Suppression of oxidative phosphorylation in tumor cells has been shown to enhance the efficacy of PD-1 immunotherapy (Noman et al., 2014; Chen et al., 2020). Furthermore, the presence of heightened levels of fatty acids in the TME can induce the accumulation of lipid droplets within immune cells, thereby commonly inducing immunosuppression (Kumagai et al., 2020; He et al., 2021). The findings of this study provide evidence that the expression of ARL4C exhibits a significant negative correlation with oxidative phosphorylation and fatty acid metabolic pathways, as depicted in Figure 4. As a result, it is postulated that ARL4C may exert an influence on the progression of cancer through the regulation of these immune-related signaling pathways. The observation that heightened ARL4C expression is linked to immune activation in cancer may offer promising prospects for ARL4C-targeted immunotherapy.

Within the pan-carcinoma landscape, the expression of ARL4C is highly correlated with immune signaling pathways, as demarcated by the red module. This correlation suggests that ARL4C is instrumental in the regulation of immune cell signaling, inflammation, and immune escape, collectively comprising critical components of the immune response. Additionally, the blue module encompasses pathways that pertain to cellular adhesion and distal metastasis. These findings implicate ARL4C as a significant contributor to both the oncogenesis and metastatic dissemination of tumor cells.

### 3.5 ARL4C expression displayed a significantly association with tumor-associated macrophages and fibroblasts

**FIGURE 5:**
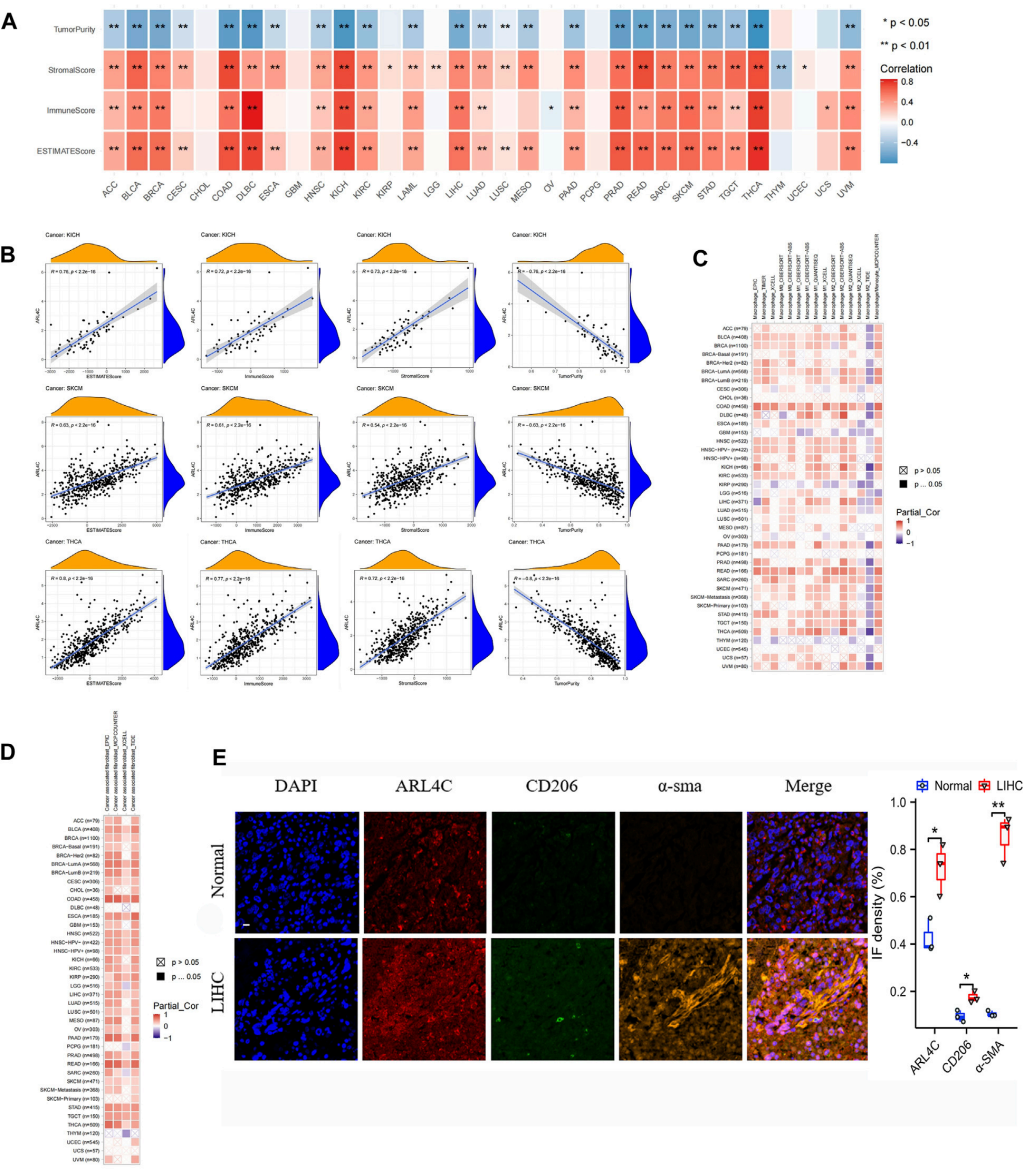
Analysis of ARL4C and tumor immune microenvironment. **(A)** The correlation between ARL4C and tumor purity, stromal score, immune score, and ESTIMATE score, as evaluated by the ESTIMATE algorithm in pan-cancer, was assessed using Spearman tests (*p < 0.05, **p < 0.01). **(B)** Scatter plots of ARL4C versus ESTIMATE algorithm assessment in KICH, SKCM, and THCA tumors by Spearman tests. **(C, D)** Spearman tests were employed to investigate the correlation between ARL4C and macrophage and fibroblast infiltration in pan-cancer. **(E)** Representative immunofluorescence staining images of tumors and normal tissues obtained from patients with liver hepatocellular carcinoma (LIHC) post-surgery are presented in this study. The staining protocol involved the use of specific markers, with blue representing DAPI, orange representing the CAF marker α-SMA, green representing the M2 macrophage marker CD206, and red representing ARL4C. The scale bar in the images corresponds to 100μm, and the experiments were conducted in triplicate (*n* = 3).

The TME serves as a key point for the survival of tumor cells and plays a significant role in various biological processes, including tumor progression, metastasis, and drug resistance. In this study, we employed the ESTIMATE algorithm to examine the relationship between the expression of ARL4C and the composition of the TME (Vitale et al., 2019; Baldominos et al., 2022). Our research results indicate a significant correlation between the expression of ARL4C and stromal scores among 28 tumors (*p* < 0.05), with a positive correlation observed in 27 tumors, except for a negative correlation with the stromal score of THYM (Figure 5A). A strong correlation exists between ARL4C and immune score across 22 tumor samples (*p* < 0.05), with positive correlations observed in 21 tumors, except for a negative correlation with the immune score of OV. Meanwhile, scatter plots were provided for three specific tumors, namely KICH, SKCM, and THCA (Figure 5B). Utilizing ssGSEA, the expression of ARL4C was categorized into high and low expression groups. Notably, high ARL4C expression demonstrated positive associations with immune pathways across plenty of tumors, including KICH, PRAD, THCA, rectum adenocarcinoma (READ), lymphoid neoplasm diffuse large B-cell lymphoma (DLBC), sarcoma (SARC), BRCA, and BLCA, among others. Conversely, high ARL4C expression only in THYM, OV, UCEC, and lung squamous cell carcinoma (LUSC) exhibited a negative association with the immune pathway (Supplementary Figure S3).

The investigation of cancer-associated fibroblasts (CAFs) and macrophages as immune cells in cancer immunotherapy has been undertaken (Costa et al., 2018; Buechler et al., 2021; Davidson et al., 2021). Consequently, we conducted Spearman correlation analysis employing various algorithms utilizing the pan-cancer macrophage and CAFs infiltration data derived from the TIMER2.0 database, with the outcomes presented in Figures 5C, D. The algorithms revealed a significant and positive correlation between ARL4C expression and TAMs and CAFs, particularly in COAD and READ, thereby highlighting the importance of ARL4C in the immunomodulatory process. Tumor (LIHC) and normal tissue specimens were collected from clinical postoperative procedures. Initially, we confirmed the presence of immune infiltration in LIHC using data from the TCGA database. Our analysis revealed that macrophages exhibited the strongest correlation with ARL4C expression in LIHC (Supplementary Figure S4A). Subsequently, we observed a significant positive correlation between the expression of CD206, a biomarker for M2 macrophages, and α-SMA, a biomarker for cancer-associated fibroblasts (CAFs), with the expression of ARL4C in LIHC based on TCGA database analysis (Supplementary Figures S4B, C). The specimens obtained from clinical procedures were subjected to immunofluorescence staining in order to corroborate the aforementioned data. The results obtained were in line with the observation that the expression of ARL4C exhibited a substantial and positive correlation with TAMs and CAFs, as depicted in Figure 5E. Additionally, this result provided further confirmation of the strong association between ARL4C and immune cells, specifically TAMs, and CAFs, thereby offering significant clues for the investigation of immune regulation pertaining to ARL4C.

### 3.6 ARL4C was also expression on different immune cells by single-cell analysis

**FIGURE 6:**
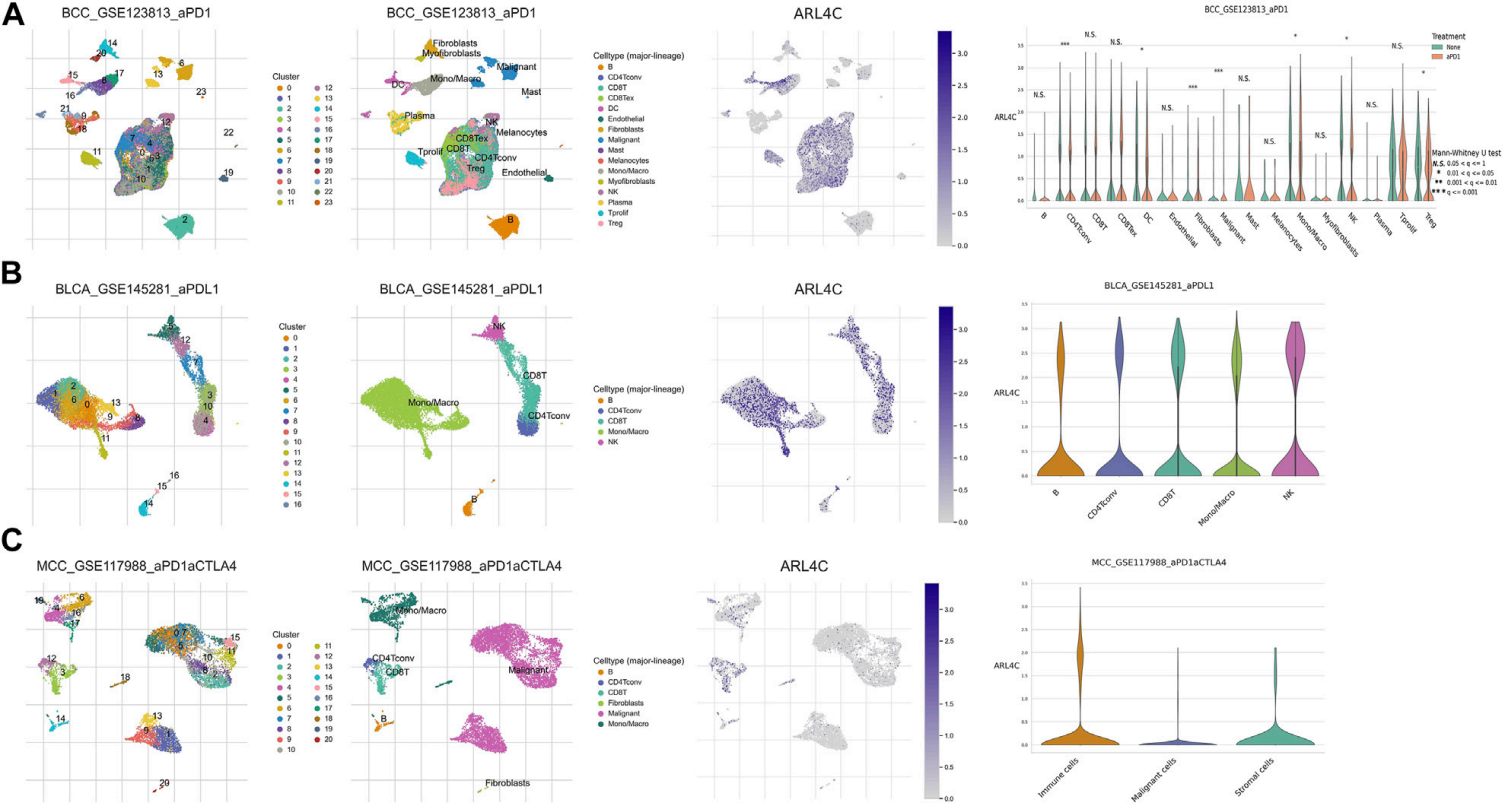
ARL4C-associated single-cell analysis. **(A)** ARL4C-associated single-cell analysis in the BCC_GSE123813. **(B)** ARL4C-associated single-cell analysis in the BLCA_GSE145281. **(C)** ARL4C-associated single-cell analysis in the MCC_GSE117988 datasets.

To explore potential mechanisms underlying the tumor immune microenvironment, we utilized a publicly available scRNA-seq (single-cell RNA-seq) dataset to investigate the expression of ARL4C across various immune cell types. Our analysis encompassed three distinct cancer samples. In the BCC_GSE123813 dataset (Figure 6A), ARL4C exhibited widespread expression on immune cells such as DC cells, monocytes, and macrophages within the TME of basal cell carcinoma (BCC). Similarly, in the BLCA_GSE145281 (Figure 6B) and MCC_GSE117988 (Figure 6C) datasets, ARL4C predominantly manifested in immune cells.

Based on our preliminary findings, there was a notable variance in ARL4C expression between malignant cells and macrophages within certain tumor types, such as the BCC immunotherapy cohort, when comparing the treated cohorts to their untreated counterparts (Figure 6A). The elevated expression of ARL4C in Tregs may indicate its involvement in the modulation of immune suppression and tolerance, thereby laying a theoretical foundation for the exploration of ARL4C in immune regulation and its potential as a predictive marker for immunotherapy research.

In the tumor microenvironment, tumor-associated macrophages (TAMs) and dendritic cells (DCs) are frequently influenced and domesticated by tumor cells to exert immunosuppressive functions, aiding tumors in evading host immune surveillance. Tregs are able to release a plethora of anti-inflammatory or immunosuppressive cytokines that suppress the activation and expansion of effector T cells. The increased expression of ARL4C in Tregs, DCs, and Treg subsets indicates its participation in the modulation of immunosuppression and tolerance. These results established a theoretical basis for investigating the role of ARL4C in immune modulation and its potential as a prognostic marker for the study of immune therapies.

### 3.7 ARL4C exhibited co-expression with immune-related genes

**FIGURE 7:**
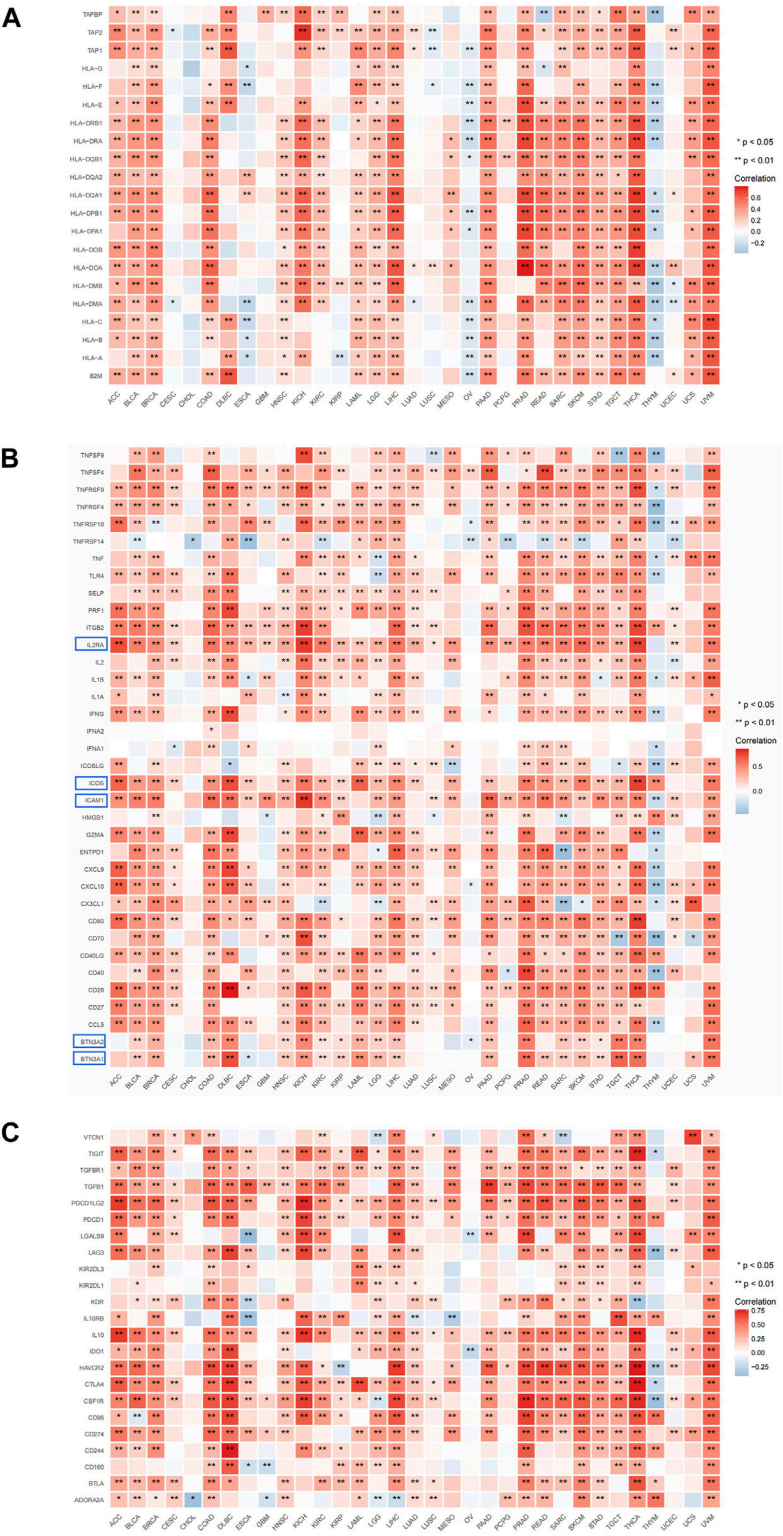
ARL4C and immune-related gene analysis. **(A)** Correlation of ARL4C with MHC. **(B)** Correlation of ARL4C with immunostimulatory checkpoints. **(C)** Correlation of ARL4C with immunosuppressive checkpoints (*p < 0.05, ***p < 0.01). Blue modules: most significant expression.

The present study employed gene co-expression analysis to investigate the association between ARL4C expression and MHC genes, as well as immunostimulatory/suppressive checkpoints, across various cancer types. The findings revealed a predominant co-expression of ARL4C with MHC genes in the majority of cancer types, wherein ARL4C exhibited a positive correlation with most MHC genes specifically in KICH, LIHC, PRAD, THCA, and UVM (Figure 7A). Conversely, ARL4C displayed a negative correlation with certain MHC genes in ESCA, OV, and THYM. Furthermore, ARL4C exhibited a positive association with immunostimulatory checkpoint markers in most cancer types, and a negative association observed with TNFRSF14 in BLCA, CHOL, ESCA, KIRC, OV, PCPG, READ, SKCM, and UCEC (Figure 7B). ARL4C exhibited a positive correlation with various immunosuppressive genes, namely TIGIT, TGFBR1, TGFB1, PDCD1LG2, PDCD1, LAG3, IL10, IDO1, HAVCR2, CTLA4, CSF1R, CD96, CD274, CD244, BTLA, and ADORA2A, across multiple cancer types including ACC, BRCA, and COAD (Figure 7C). Conversely, ARL4C displayed a negative association with immunosuppressive genes in certain cancer types. For instance, in OV, ARL4C exhibited a negative correlation with LGALS9 and IDO1. Similarly, in THYM, ARL4C demonstrated a negative correlation with TIGIT, LAG3, HAVCR2, CTLA4, and CSF1R.

### 3.8 The correlation between ARL4C expression and immunotherapy response or drug sensitivity

**FIGURE 8:**
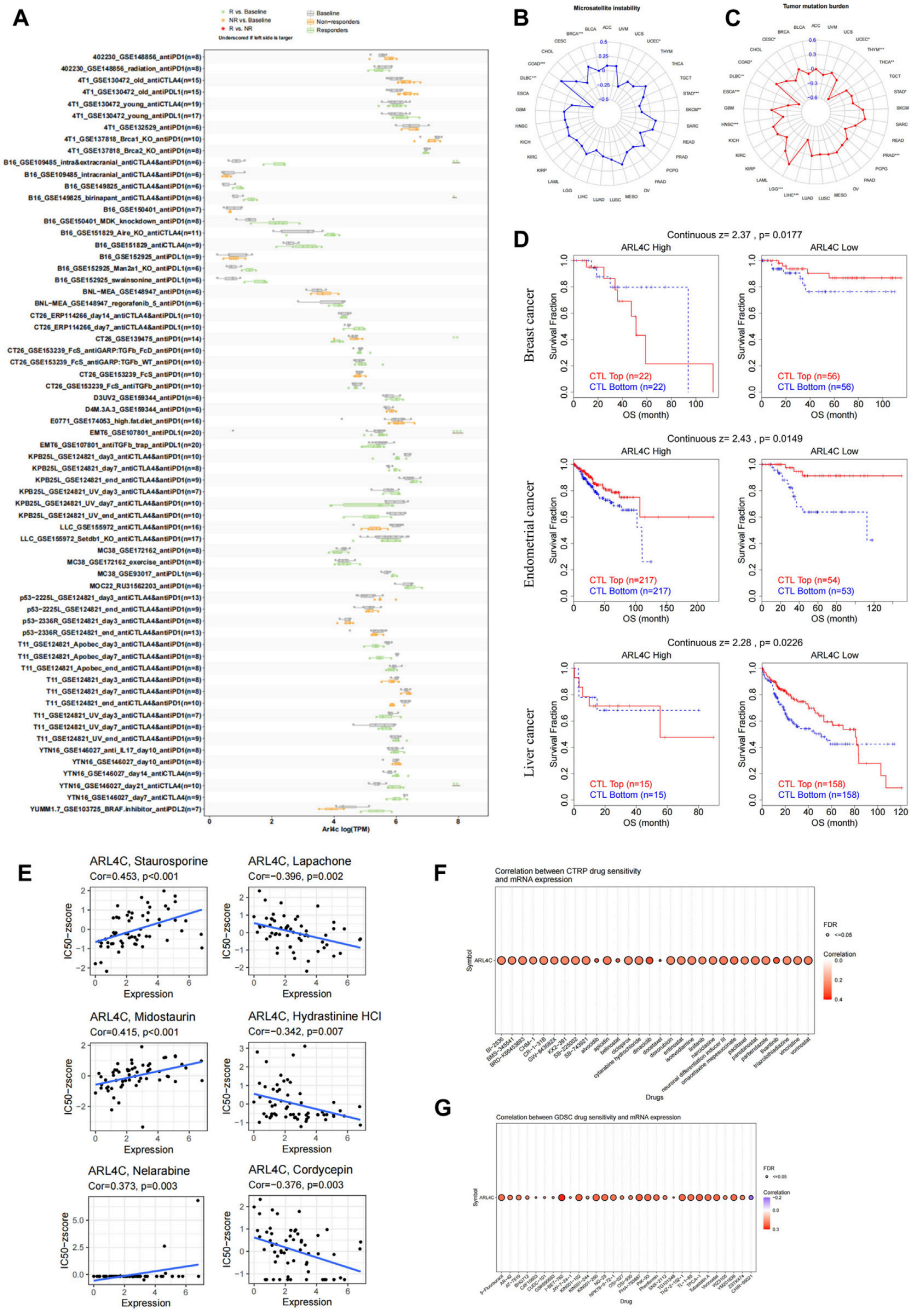
Correlation of ARL4C expression with the immunotherapeutic response and with drug sensitivity. **(A)** The predictive analysis of ARL4C-related immunotherapy at the animal level was conducted using the TISMO database. Statistical evaluation of the differences between groups was performed using the Wald test with DESeq2, with significance levels denoted as *p < 0.05, **p < 0.01, ***p < 0.001. **(B, C)** The Spearman correlation coefficient analysis was conducted to examine the relationship between ARL4C expression and MSI and TMB. **(D)** The TIDE assessment was used to evaluate the relationship between ARL4C and CTL dysfunction. **(E)** The relationship between ARL4C expression and drug sensitivity was investigated. **(F, G)** The correlation between CTRP and GDSC drug sensitivity and mRNA expression was examined by Spearman correlation analysis.

Immunotherapy utilizing immune checkpoint blockers demonstrates promising potential in the field of oncology treatment. However, it is important to acknowledge that the subset of patients who benefit from this therapy, which enhances the body’s anti-tumor immunity, is limited (Chen et al., 2018; Jiang et al., 2018). Consequently, it is crucial to investigate sensitive and stable biomarkers that can predict the response to immunotherapy, in order to select appropriate clinical interventions. In this study, we assessed the predictive value of ARL4C in immunotherapy by analyzing data from the B16_GSE109485, B16_GSE149825, EMT6_GSE107801, YTN16_GSE146029, and CT26_GSE139475 databases (Figure 8A). With the exception of CT26_GSE139475, which exhibited a greater number of non-responders compared to the baseline, the remaining groups demonstrated a higher proportion of responders than the baseline. *In vitro* experiments, we investigated the expression levels of ARL4C in four tumor cell lines treated with various cytokines, namely IFNβ, IFNγ, TGFβ, and TNFα. The results revealed significant disparities in ARL4C expression within the IFNβ and IFNγ-treated tumor cell lines (Supplementary Figure S5). It is plausible that immunotherapy may exhibit enhanced efficacy in patients with elevated TMB/MSI (Huang et al., 2022; Tang et al., 2022). Our research findings indicate a significant correlation between ARL4C and MSI in six different tumor types. Specifically, ARL4C exhibited a positive correlation with BRCA and COAD, while showing a negative correlation with DLBC, SKCM, stomach adenocarcinoma (STAD), and UCEC (Figure 8B). Additionally, we observed a significant correlation between ARL4C and TMB in 12 tumor types. This correlation was reflected in a positive association with COAD and LGG, and a negative association with CESC, DLBC, ESCA, head and neck squamous cell carcinoma, LIHC, PRAD, STAD, THCA, THYM, and UCEC (Figure 8C).

Multiple studies have indicated that cytotoxic T lymphocytes (CTLs) are crucial in antitumor immunity, and that dysfunction of these CTLs can contribute to tumor immune evasion and resistance to immunotherapy (Zarour, 2016; Jiang et al., 2018; Mami-Chouaib et al., 2018). In this study, we investigated the potential correlation between ARL4C and CTL dysfunction, employing the TIDE calculation scheme (Figure 8D). Our findings reveal a notable association between ARL4C expression and CTL dysfunction in breast, endometrial, and liver cancer (*p* = 0.0177, *p* = 0.0149, *p* = 0.0226). In the context of diminished ARL4C expression, an elevated infiltration of CTLs was associated with prolonged survival among patients, while heightened ARL4C expression levels mitigated or even reversed these advantageous outcomes.

Our study sought to investigate the responsiveness of ARL4C to drugs by utilizing various drug databases and to examine the association between ARL4C expression and FDA-approved drug sensitivity through the utilization of the CellMiner database. Notably, the expression level of ARL4C exhibited positive correlations with Staurosporine, Midostaurin, and Nelarabine (Figure 8E). Cordycepin has been documented to exhibit anti-tumor effects through the facilitation of apoptosis and autophagy, as well as the inhibition of CD47 expression to augment anti-tumor immunity. Analysis of the CTRP (Figure 8F) and GDSC database (Figure 8G) revealed a consistent positive correlation between ARL4C expression and all drugs, except for CHIR-99021. These findings offer novel perspectives on treatment strategies by predicting the association between ARL4C expression and drug response.

## 4 Discussion

ARL4C assumes critical functions in the biology of tumor cells, encompassing stem cell-like characteristics, proliferation, and resistance to therapeutic agents. Our findings demonstrate that ARL4C exhibits extensive localization within the plasma membrane and cytosol across various tumor cell lines, suggesting that its broad distribution may be a fundamental prerequisite for its functional roles. We observed a diverse distribution pattern of ARL4C in multiple anatomical regions, namely the spleen, cerebellar hemisphere, and sun-exposed skin on the lower legs. Notably, elevated levels of ARL4C were detected in THYM, CHOL, and OV, surpassing the expression levels observed in normal tissues. Furthermore, our analysis revealed that ARL4C expression was significantly higher in 23 out of 33 cancer types examined, suggesting a potential comprehensive role of ARL4C in tumorigenesis. To validate these findings, we conducted IHC experiments to confirm the expression of ARL4C.

We identified a high frequency of ARL4C alterations in sarcoma, brain lower grade glioma, and esophageal adenocarcinoma, emphasizing the critical role of mutations in the progression of tumorigenesis. The findings of the study revealed a positive correlation between the expression level of ARL4C and the methylation of its promoter in BRCA, COAD, KIRP, and LIHC. Conversely, a negative correlation was observed in TGCT, UCEC, LUSC, and KIRC. Zhang et al. conducted a study that demonstrated the inhibitory effects of knockdown of ARL4C on various EMT phenomena, such as proliferation, migration, and invasion, in kidney cancer cell lines (Zhang et al., 2022). They also found that this inhibition was regulated by the Wnt/β-Catenin pathway.

The TME plays a significant role in various stages of tumor initiation, progression, metastasis, and drug resistance, acting as a facilitator in cancer development. ARL4C, a member of the ARL4 protein family, is involved in the formation of epithelial tubular structures during organ development in normal physiological conditions. However, in pathological conditions, it contributes to tumorigenesis, growth, and other related processes. Concurrently, ARL4C in ovarian cancer may be classified as a tumor suppressor due to its high expression to impede cell migration. We contend that the existing research fails to fully elucidate the intricate mechanism of ARL4C in various tumors and its association with the tumor immune microenvironment. Consequently, we conducted a bioinformatics analysis of ARL4C across multiple cancer types and investigated its underlying impact on tumor immunology. Our evaluation revealed a statistically significant increase in ARL4C mRNA expression in 23 different tumor tissues compared to normal tissue. We also observed a positive correlation between the upregulation of ARL4C expression and poor prognosis in various cancer types, including BLCA, GBM, KIRC, KIRP, LGG, OV, PAAD, UCEC, and UVM. These findings align with previous research, indicating that ARL4C holds promise as a prognostic biomarker in oncology. Based on the analysis of publicly accessible scRNA datasets, it was observed that ARL4C primarily exhibits expression in immune cells, including CD4+T lymphocytes, CD8+T lymphocytes, NK cells, monocytes, and macrophages. Previous studies have demonstrated the significance of NK cells and CD8+T lymphocytes as pivotal immune cells in the TME, which can be activated upon the release of Th1 cytokines (Budi and Farhood, 2023). Our results further strengthen the association between ARL4C and the TME.

Furthermore, the ESTIMATE algorithm effectively and efficiently predicts tumor purity and reflects the characteristics of the tumor microenvironment. Our results revealed a significant correlation between the expression of ARL4C and stromal scores among 28 tumors (*p* < 0.05), with a positive correlation observed in 27 tumor types. There was a significant correlation between ARL4C expression and the immune scores of 22 types of tumors (*p* < 0.05), among which it was positively correlated with 21 tumors. Furthermore, ARL4C was found to be significantly associated with various immune-related pathways, including TNFα-NFκB, INF-γ, INF-α and IL6-JAK-STAT3. INF-γ, which is the sole type II interferon, serves as a key cytokine for numerous cells and plays a role in modulating tumor immunity (Wang et al., 2019; Neo et al., 2020; Nakamura et al., 2021; Liao et al., 2022). Additionally, the IL6-JAK-STAT3 pathway is involved in immune regulation, lymphocyte growth, and differentiation (Dambal et al., 2020; Peng et al., 2021). The findings of this study provide further evidence supporting the association between ARL4C and immune regulation in these specific types of cancers. Additionally, analysis of the TIMER2.0 database revealed a positive correlation between ARL4C and the infiltration of various immune cells, such as macrophages and CAFs. TAMs as a major part of the TME are associated with the progression of tumors. TAM can be classified as M1 and M2 type co-existing in the TME. On account of its plasticity, TAMs can switch from one type to another depending on the environment they reside (Vitale et al., 2019). Interestingly, ARL4C was found to be highly expressed in the M2 TAM of LIHC. However, both TNF responsiveness and IFN-α/γ responsiveness are positively correlated with ARL4C expression, which indicates an M1-preferred milieu. This situation is not only present in this study. Surprisingly, Müller et al. (2017) indicated that TAMs frequently co-express M1 and M2 type genes in individual cells which enhanced the difficulty of taking them apart, which may provide evidence of the existence of the intermediate state of TAMs. Immunofluorescence staining of tumor and normal tissue specimens further confirmed a positive correlation between ARL4C and TAMs and CAFs, suggesting a close relationship between ARL4C and immune infiltration in tumor cells. Furthermore, our investigation demonstrated that ARL4C was linked to TMB expression in 12 different types of cancers. In a separate study, Wang et al. identified TNFRSF14 as a protective marker involved in the proliferation of bladder cancer (BLCA) cells (Wang et al., 2021). Additionally, Carreras et al. confirmed a strong association between TNFRSF14 and poor prognosis in follicular lymphoma and ESCA (Carreras et al., 2019). Based on these findings, it is justifiable to consider promoting ARL4C as a potentially predictive target for immunotherapy in the treatment of these tumors.

Ultimately, our findings indicate a positive association between the expression level of ARL4C and the sensitivity of tumors to various drugs, such as Staurosporine, Midostaurin, and Nelarabine. Specifically, Staurosporine inhibits T-cell activation, proliferation, and cytokine production in a manner that is dependent on dosage (Weichsel et al., 2008). Additionally, the impact of Midostaurin on the TME influences the efficacy of anti-PD-1 therapy for colon cancer treatment (Lai et al., 2022). Furthermore, Nelarabine, a purine nucleoside analog, is commonly employed in the management of T-cell malignancies (Agrawal et al., 2021). In contrast, the expression of ARL4C exhibited a negative correlation with Lapachone, Hydrastinine HCL, and Cordcepin. NQO1, which is highly enriched in tumors, plays a pivotal role in the generation of reactive oxygen species (ROS) through the activation of β-lapachone. The accumulation of ROS disrupts the redox balance in tumor cells, ultimately leading to cell death. Conversely, β-lapachone stimulates the immune response by inducing the release of HMGB1 (high mobility group box 1) (Flick et al., 2013; Li et al., 2019). Additionally, Cordycepin has been reported to exert anti-tumor effects by promoting apoptosis and autophagy, as well as suppressing CD47 expression to enhance anti-tumor immunity. In both the CTRP and GDSC databases, it was observed that ARL4C expression exhibited a positive correlation with most drugs. For instance, the CDK inhibitor Dinaciclib was found to enhance anti-PD1-mediated suppression in solid tumor therapy (Hossain et al., 2018). Furthermore, Dinaciclib was shown to enhance NK cytotoxicity in the treatment of acute granulocytic leukemia (Yun et al., 2019). Additionally, I-BET 762 demonstrated the ability to reduce c-Myc and p-Erk 1/2 protein levels, inhibit cancer cell proliferation, and suppress the generation of multiple inflammatory cytokines (Leal et al., 2017). These findings suggest that assessing ARL4C expression and drug response could offer a novel therapeutic strategy. ARL4C-targeted therapy and prediction hold promise as a potential approach for tumor treatment.

In summary, our study extensively investigated the predictive value and immune-related implications of ARL4C in pan-cancer by bioinformatics tools. The mechanism was further validated through experimental techniques such as IHC and IF. However, it is important to note that the scope of our molecular biology experiments was not exhaustive, prompting further exploration into the underlying mechanism of ARL4C in greater detail. Fortunately, our findings confirmed the involvement of ARL4C in the tumor immune microenvironment, thereby expanding the potential of ARL4C-targeted tumor immunotherapy.

## 5 Conclusion

The expression level of ARL4C may play an important role in cancer development and poor prognosis. The immunotherapy drug susceptibility suggested ARL4C might be a novel immunotherapeutic target in the future.

## Data Availability

The original contributions presented in the study are included in the article/Supplementary Material, further inquiries can be directed to the corresponding author.
