# Supplementary material for: The role of ARL4C in predicting prognosis and immunotherapy drug susceptibility in pan-cancer analysis
Source: Front Pharmacol. 2023 Dec 20;14:1288492. doi: 10.3389/fphar.2023.1288492 (PMC10765536; doi:10.3389/fphar.2023.1288492)
Supplement: Supplementary file 2 [file Image5.PDF]

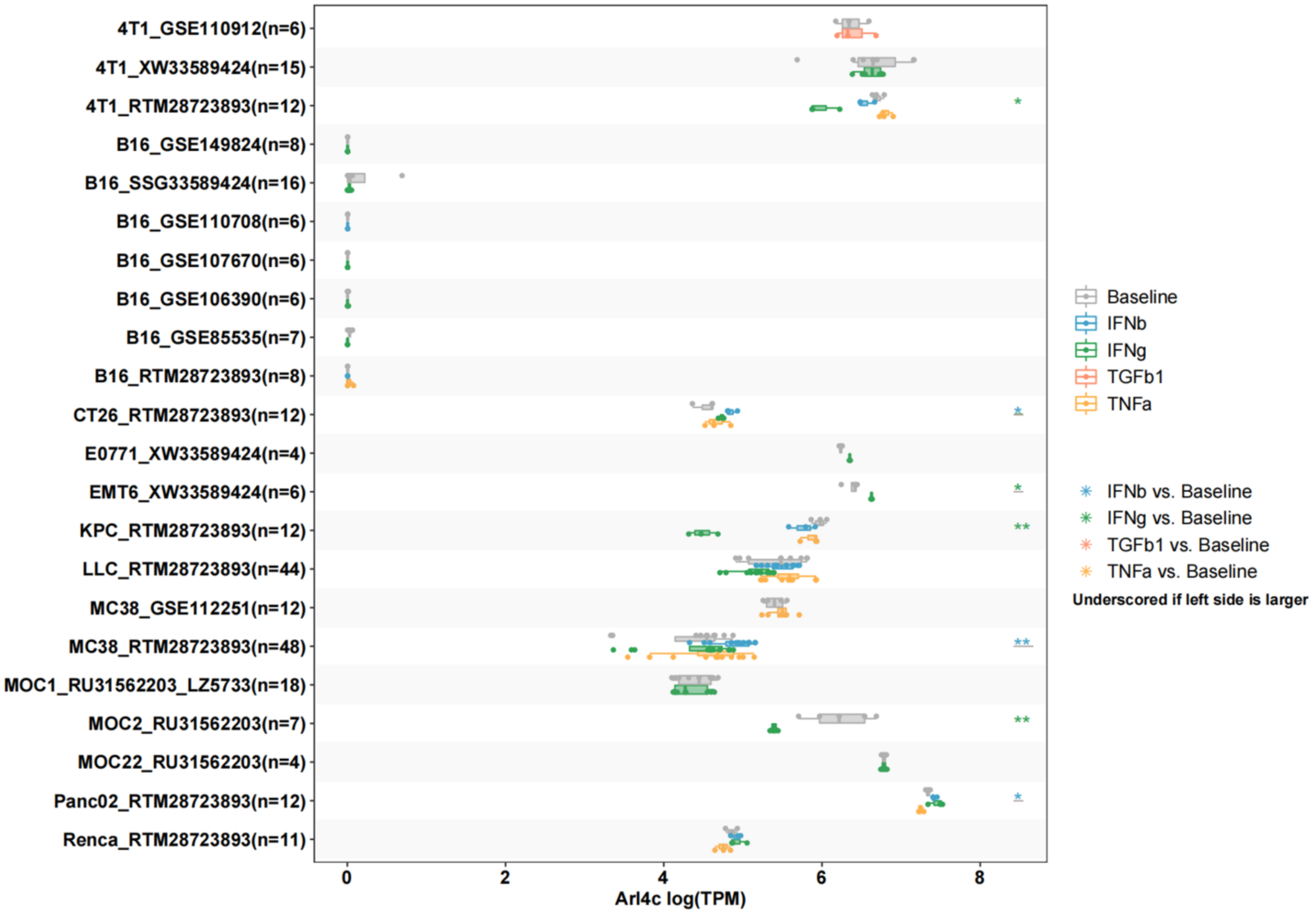

Supplementary Figure 5. In vitro experiments to explore the expression level of ARL4C in four cytokine-treated tumor cell lines including IFNβ, IFNγ, TGFβ, and TNFα.
