# Supplementary material for: The role of ARL4C in predicting prognosis and immunotherapy drug susceptibility in pan-cancer analysis
Source: Front Pharmacol. 2023 Dec 20;14:1288492. doi: 10.3389/fphar.2023.1288492 (PMC10765536; doi:10.3389/fphar.2023.1288492)
Supplement: Supplementary file 3 [file Table1.PDF]

Supplementary Table 1 The Single gene logistics regression analysis of ARL4C

Cancer types	Characteristics	Total (N)	OR (95% CI)	P value
BLCA				
	Pathologic T stage (T3&T4 vs. T1&T2)	378	2.565 (1.643 - 4.003)	**< 0.001**
	Pathologic N stage (N1&N2&N3 vs. N0)	368	1.862 (1.204 - 2.878)	**0.005**
	Pathologic M stage (M1 vs. M0)	212	2.760 (0.782 - 9.737)	0.115
	Age (> 70 vs. <= 70)	412	1.082 (0.733 - 1.597)	0.691
KIRP				
	Pathologic T stage (T3&T4 vs. T1&T2)	289	0.974 (0.555 - 1.708)	0.927
	Pathologic N stage (N1&N2 vs. N0)	78	3.200 (1.219 - 8.398)	0.018
	Pathologic M stage (M1 vs. M0)	104	2.130 (0.503 - 9.020)	0.304
	Age (> 60 vs. <= 60)	288	0.494 (0.309 - 0.791)	0.003
UCEC				
	Clinical stage (Stage III&IV vs. Stage I&II)	554	2.521 (1.718 - 3.700)	< 0.001
	Tumor invasion(%) (>= 50 vs. < 50)	476	1.081 (0.754 - 1.552)	0.671
	Age (> 60 vs. <= 60)	551	1.143 (0.809 - 1.614)	0.448
COAD				
	Pathologic T stage (T3&T4 vs. T1&T2)	477	1.719 (1.086 - 2.723)	0.021
	Pathologic N stage (N1&N2 vs. N0)	478	1.749 (1.209 - 2.530)	0.003
	Pathologic M stage (M1 vs. M0)	415	1.132 (0.669 - 1.916)	0.645
	Age (> 65 vs. <= 65)	478	0.812 (0.563 - 1.170)	0.264
SKCM				
	Pathologic T stage (T3&T4 vs. T1&T2)	365	0.743 (0.480 - 1.151)	0.183
	Pathologic N stage (N1&N2&N3 vs. N0)	415	1.362 (0.923 - 2.012)	0.120
	Pathologic M stage (M1 vs. M0)	444	0.775 (0.344 - 1.746)	0.538
	Age (> 60 vs. <= 60)	464	0.934 (0.648 - 1.347)	0.716
LGG				
	WHO grade (G3 vs. G2)	469	2.428 (1.675 - 3.521)	< 0.001
	IDH status (Mut vs. WT)	528	0.021 (0.007 - 0.067)	< 0.001
	Age (> 40 vs. <= 40)	531	2.061 (1.459 - 2.913)	< 0.001
