# Supplementary material for: The role of ARL4C in predicting prognosis and immunotherapy drug susceptibility in pan-cancer analysis
Source: Front Pharmacol. 2023 Dec 20;14:1288492. doi: 10.3389/fphar.2023.1288492 (PMC10765536; doi:10.3389/fphar.2023.1288492)
Supplement: Supplementary file 4 [file Image4.PDF]

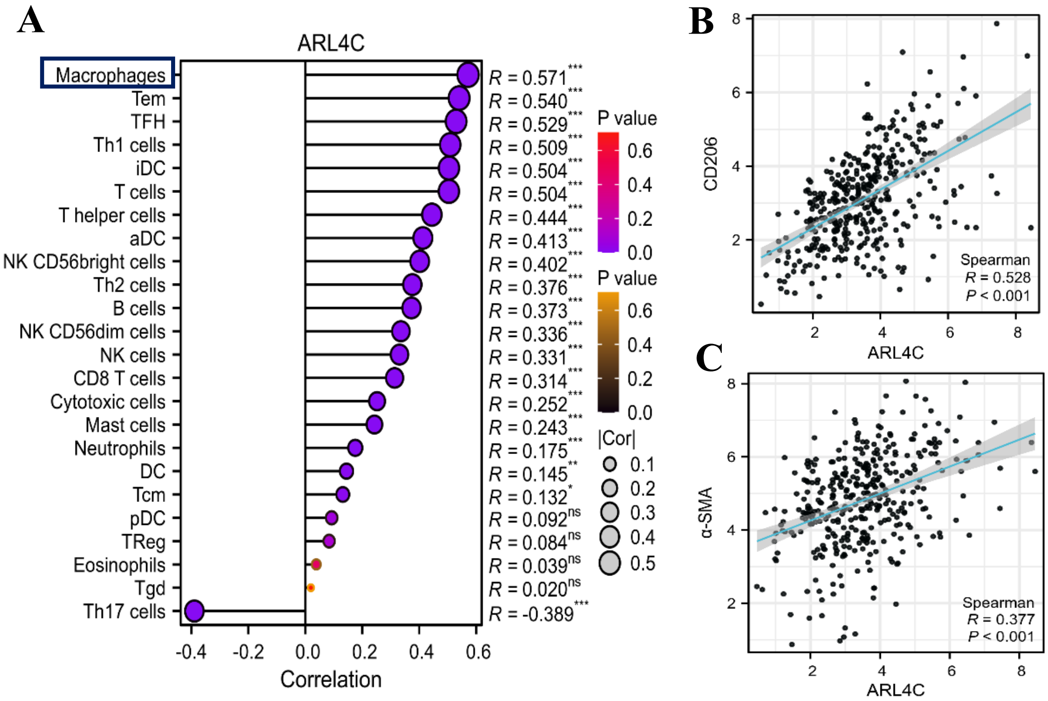

Supplementary Figure 4 The immune filtration of LIHC based on TCGA database. (A) The correlations of different immune cells of LIHC. (B) The significantly positive correlation between CD206, the biomarker of M2 macrophages, and ARL4C expression of LIHC. (C) The significantly positive correlation between α-SMA, the biomarker of cancer-associated fibroblasts, and ARL4C expression of LIHC.
