# Supplementary figures and images for: The role of ARL4C in predicting prognosis and immunotherapy drug susceptibility in pan-cancer analysis
Source: Front Pharmacol. 2023 Dec 20;14:1288492. doi: 10.3389/fphar.2023.1288492 (PMC10765536; doi:10.3389/fphar.2023.1288492)

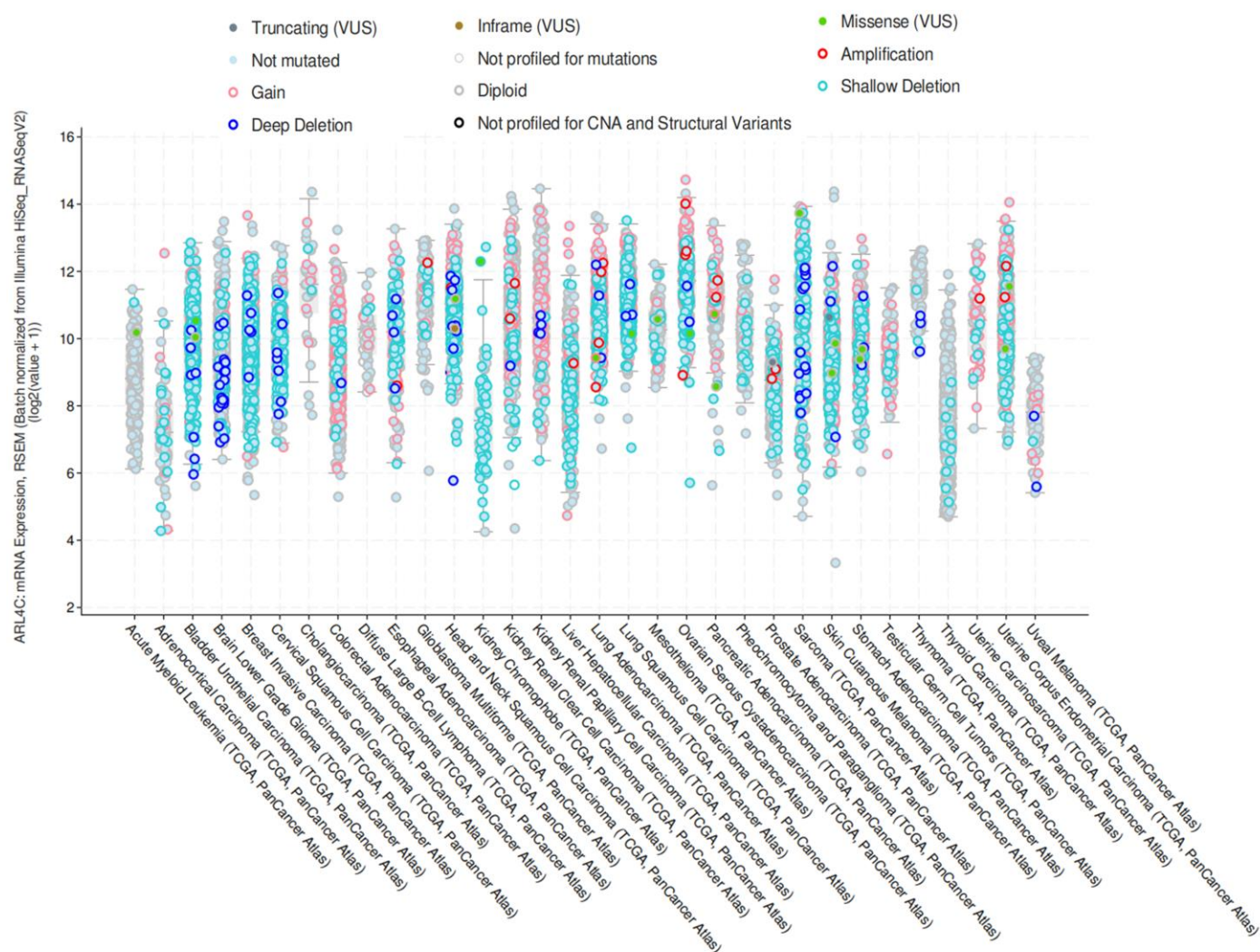

**Supplementary Figure 2.** Mutation counts of ARL4C in cancer.

Supplement: Supplementary file 5 [file Image2.PDF]

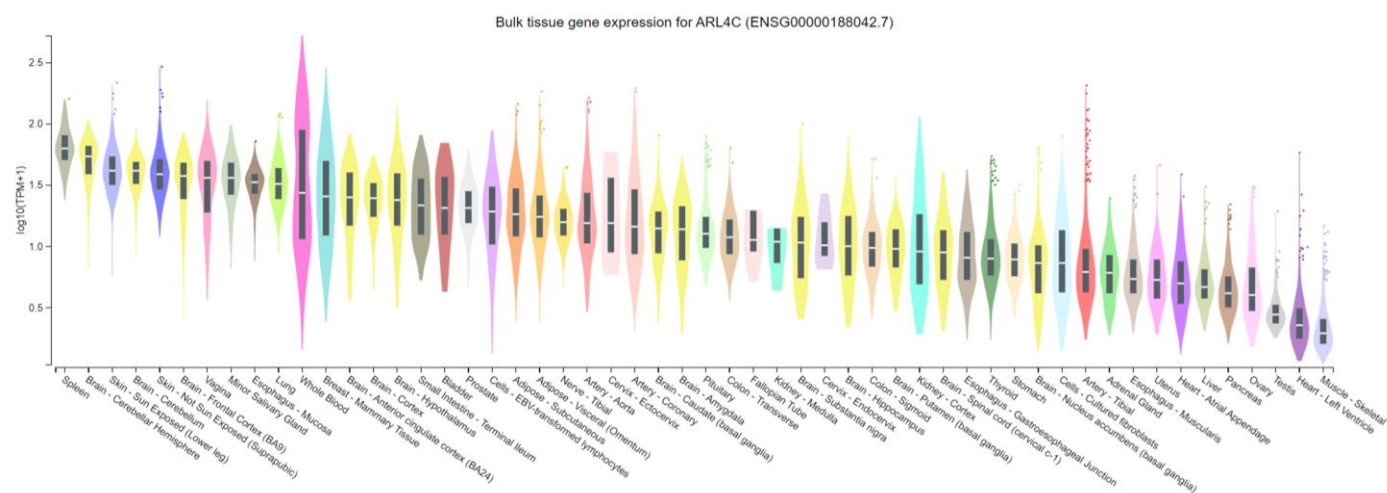

**Supplementary Figure 1.** Expression of ARL4C mRNA in normal tissues.

Supplement: Supplementary file 7 [file Image1.PDF]
