# Supplementary material for: The role of ARL4C in predicting prognosis and immunotherapy drug susceptibility in pan-cancer analysis
Source: Front Pharmacol. 2023 Dec 20;14:1288492. doi: 10.3389/fphar.2023.1288492 (PMC10765536; doi:10.3389/fphar.2023.1288492)
Supplement: Supplementary file 6 [file Image3.PDF]

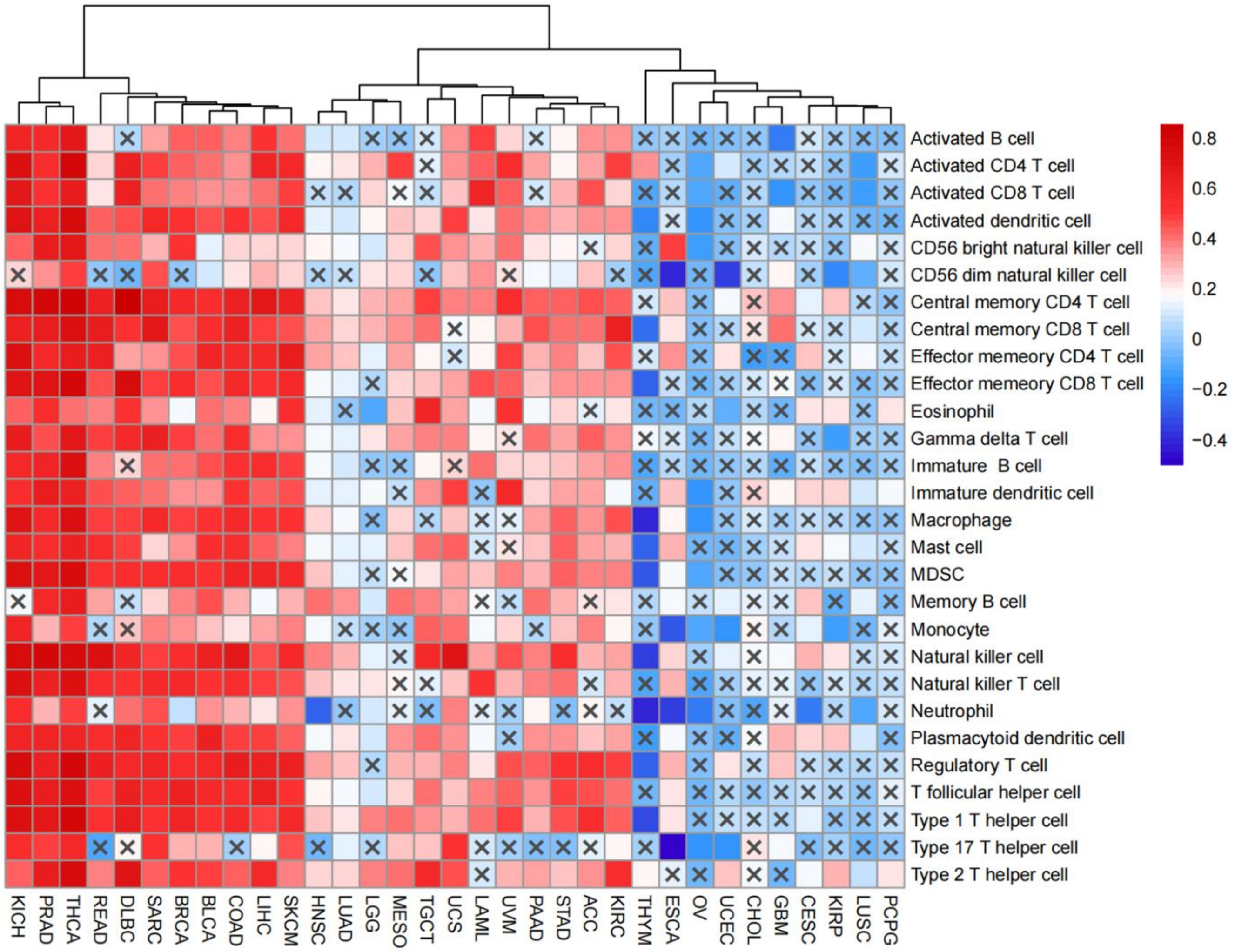

Supplementary Figure 3. ARL4C was categorized into two groups of high and low expression, and their immune cell activities in different cancers.
